# Supplementary material for: Stereotyped terminal axon branching of leg motor neurons mediated by IgSF proteins DIP-α and Dpr10
Source: eLife. 2019 Feb 4;8:e42692. doi: 10.7554/eLife.42692 (PMC6391070; doi:10.7554/eLife.42692)
Supplement: Supplementary file 1. [file elife-42692-supp1.docx]

**Supplementary File 1.** List of *DIP* and *dpr* MiMIC lines used to generate *T2A-Gal4* lines

| ***DIP*** | **MiMIC #** |
| --- | --- |
| *DIP-α* | MI02031 |
| *DIP-β* | MI01971 |
| *DIP-δ* | MI08287 |
| *DIP-ε* | MI11827 |
| *DIP-η* | MI07948 |
| *DIP-γ* | MI03222 |
| *DIP-θ* | MI03191 |
| *DIP-ζ* | MI03838 |

| ***dpr*** | **MiMIC #** |
| --- | --- |
| *dpr-1* | MI02201 |
| *dpr-2* | MI02530 |
| *dpr-3* | MI05963 |
| *dpr-4* | MI08665 |
| *dpr-5* | MI11085 |
| *dpr-6* | MI04582 |
| *dpr-7* | MI05719 |
| *dpr-8* | MI11830 |
| *dpr-9* | MI03594 |
| *dpr-10* | MI03557 |
| *dpr-11* | MI01743 |
| *dpr-12* | MI01695 |
| *dpr-13* | MI05577 |
| *dpr-15* | MI01408 |
| *dpr-16* | MI05173 |
| *dpr-17* | MI08707 |
