# Supplementary material for: Stereotyped terminal axon branching of leg motor neurons mediated by IgSF proteins DIP-α and Dpr10
Source: eLife. 2019 Feb 4;8:e42692. doi: 10.7554/eLife.42692 (PMC6391070; doi:10.7554/eLife.42692)
Supplement: Supplementary file 2. [file elife-42692-supp2.docx]

**Supplementary File 2. Genotypes Used**

| Fig1A | *VGlut-T2A-QF2, 10XQUAS-6XGFP* |
| --- | --- |
|  | *OK371-Gal4/10XQUAS-6XmCherry* (VK00018) *;* *20XUAS-6XGFP* (attp2)*, Mef2-QF2* |
| Fig1B-C  Fig1-fig sup.1C-G | *dpn>KDRT>Cre ; Act>LoxP>LexA, LexA-myr::GFP ; UAS-KD/10C12-Gal4* |
| Fig1-fig sup.1A,  Video1, Video2 | *dpn>KDRT>Cre ; Act>LoxP>LexA, LexA-myr::GFP/ VGlut-T2A-QF2, 10XQUAS-6XmCherry* (VK00018) *; UAS-KD/10C12-Gal4* |
| Fig2, Fig2-fig sup.1 | *MiMIC-T2A-Gal4* (Chr2/3) X *20XUAS-6XGFP (attp2)* |
| *Vglut*  Intersect. | *20XUAS-6XGFP* (VK00018)*; MiMIC-T2A-Gal4* or *MiMIC-T2A-Gal4; 20XUAS-6XGFP* (attp2) X *tub>FRT.Gal80> ; VGlut-T2A-QF2; QUAS-Flp0.2G* |
| Fig3A | *10XQUAS-6XmCherry; DIP-α-A8-Gal4* (86Fa)*/20XUAS-6XGFP* (attp2)*, Mef2-QF2* |
| Fig3B | WT: *DIP-α-A8-Gal4* (attp2)*/* *20XUAS-6XGFP* (attp2) |
|  | Mutant: *DIP-α^7-1^//;; A8-Gal4* (attp2)/*20XUAS-6XGFP* (attp2) |
|  | Rescue: *DIP-α^7-1^//;; A8-Gal4* (attp2), *UAS-DIP-α-V5* (VK00027)*/A8-Gal4* (attp2), *20XUAS-6XGFP* (86Fa) |
| Fig3C | WT: *DIP-α-A8-Gal4* (86Fa)(this insertion exhibits stochastic expression in the α-leg MNs*)/20XUAS-6XGFP* (attp2) |
|  | *DIP-α* Mutant: *DIP-α^7-1^//; hs-Flp^122.2^/ tub>FRT.Gal80>; A8-Gal4* (attp2), *A8-Gal4* (86Fa)*/20XUAS-6XGFP* (attp2) |
| Fig3D,  Fig3-fig sup.ID-F | WT/Controls:  *DIP-α-A8-Gal4* (attp2)*/* *20XUAS-6XGFP* (attp2)  *DIP-α-T2A-QF2/+;;* *10XQUAS-6XGFP* (attp2)  *DIP-α-T2A-Gal4*/+*;;* *20XUAS-6XGFP* (attp2)  *DIP-α-T2A-QF2//;; 10XQUAS-6XGFP* (attp2) |
|  | *DIP-α* Mutant:  *DIP-α^7-1^//;; A8-Gal4* (attp2)/*20XUAS-6XGFP* (attp2)  *DIP-α-T2A-Gal4/DIP-α^7-1^;; 20XUAS-6XGFP* (attp2)  *DIP-α-T2A-Gal4//;; 20XUAS-6XGFP* (attp2)  *Df(ED6712)/DIP-α-T2A-Gal4;; 20XUAS-6XGFP* (attp2) |
|  | *DIP-α* Rescue:  *DIP-α^7-1^//;; A8-Gal4* (attp2), *UAS-DIP-α-V5* (VK00027)*/A8-Gal4* (attp2), *20XUAS-6XGFP* (86Fa)  *DIP-α-T2A-Gal4/DIP-α^7-1^;; 20XUAS-6XGFP* (attp2), *UAS-DIP-α-V5* (VK00027) |
| Fig3E | MARCM: *y,w,hs-Flp^1.22^; OK371-Gal4, FRT42D/ FRT42D, tub-Gal80; 20XUAS-6XGFP* (attp2), *UAS-DIP-α-V5* (VK00027) |
|  | *hkb-Gal4*: *DIP-α^7-1^//;; 20XUAS-6XGFP (attp2), hkb-Gal4/UAS-DIP-α-V5* (VK00027) |
| Fig3-fig sup.1B | *DIP-α-GFSTF;; DIP-α-A8-Gal4* (attp2)*/20XUAS-6XmCherry* (attp2) |
| Fig3-fig sup.1C | *DIP-β^1-95^//;; 13C09-Gal4* (attp2)*/20XUAS-6XGFP* (attp2)  OK371-Gal4, 20XUAS-6XGFP (VK00018); *DIP-γ^1-67^//*  *UAS-DIP-ζ* *RNAi;* *20XUAS-6XGFP (attp2), Hkb-Gal4* |
| Fig3-fig sup.2A | *DIP-β-T2A-QF2;; 10XQUAS-6XmCherry* (attp2)*/DIP-α-A8-Gal4* (attp2), *20XUAS-6XGFP* (86Fa) |
| Fig3-fig sup.2B | *DIP-β^1-95^//;; A8-Gal4* (attp2)*/20XUAS-6XGFP* (attp2) |
|  | *DIP-α^7-1^, DIP-β^1-95^//;; A8-Gal4* (attp2)*/20XUAS-6XGFP* (attp2) |
| Fig3-fig sup.2C | *DIP-α-T2A-Gal4/DIP-α^7-1^;; 20XUAS-6XGFP* (attp2), *UAS-DIP-β* (VK00027) |
| Fig4A, Fig4-fig sup.1A | *DIP-α-T2A-QF2/y,w,hs-Flp^1.22^; OK371-Gal4, FRT42D/ FRT42D, tub-Gal80, tub-QS; 10XQUAS-6XGFP* (attp2)*/ 20XUAS-6XmCherry* (attp2) |
| Fig4B | *DIP-α-T2A-QF2; OK371-Gal4; 10XQUAS-6XGFP* (attp2)*/20XUAS-6XmCherry* (attp2) |
| Fig4C  Fig4-fig sup.1C-D | *DIP-α-GFSTF/DIP-α-T2A-QF2;; 20XUAS-6XmCherry* (attp2) |
| Fig4D-E  Fig4-fig sup.2 | *DIP-α-T2A-Gal4/DIP-α^7-1^; tub-Gal80^ts^; 20XUAS-6XGFP* (attp2)/*UAS-DIP-α-V5* (VK00027)  Positive Control: *DIP-α-T2A-Gal4/DIP-α^7-1^; 20XUAS-6XGFP* (attp2)/*UAS-DIP-α-V5* (VK00027)  Negative Control: *DIP-α-T2A-Gal4/DIP-α^7-1^; tub-Gal80^ts^; 20XUAS-6XGFP* (attp2 |
| Fig4-fig sup.1B | *DIP-α-GFSTF/DIP-α^7-1^;; DIP-α-A8-Gal4* (attp2)*/20XUAS-6XmCherry* (attp2) |
| Fig4-fig sup.1E | *DIP-α-T2A-QF2/+;;* *10XQUAS-6XGFP* (attp2) |
| Fig4-fig sup.1F | *DIP-α-T2A-Gal4*/+*;;* *20XUAS-6XGFP* (attp2), *UAS-DIP-α-V5* (VK00027) |
| Fig5A,D | *DIP-α-T2A-QF2/+; 10XQUAS-6XGFP* (VK00018)*; dpr6,10^-^//*  *DIP-α-T2A-QF2/+; 10XQUAS-6XGFP* (VK00018)*; dpr6^1-116^//*  *DIP-α-T2A-QF2/+; 10XQUAS-6XGFP* (VK00018)*; dpr10^1-29^//* |
| Fig5B,D | *DIP-α-T2A-QF2/+; UAS-dpr10-V5* (VK0002)*, 10XQUAS-6XGFP* (VK00018)*/Mef2-Gal4; dpr10^1-29^//*  *DIP-α-T2A-QF2/+; UAS-dpr6-V5* (VK0002)/*Mef2-Gal4; dpr10^1-29^, 10XQUAS-6XGFP* (VK00027)*/ dpr10^1-29^* |
| Fig5C,Fig5.fig sup.1E-F | *DIP-α-T2A-QF2/+;; dpr10-GFSTF/10XQUAS-6XmCherry* (attp2) |
| Fig5-fig sup.1A-B | *DIP-α-T2A-QF2/+;UAS-dpr10-RNAi*/*Mef2-Gal4; dpr10^1-29^, 10XQUAS-6XGFP* (VK00027)*/ +* |
| Fig5-fig sup.1A | *DIP-α-T2A-QF2/+; UAS-dpr10-V5* (VK0002)*, 10XQUAS-6XGFP* (VK00018)*/DIP-ε-T2A-Gal4; dpr10^1-29^//* |
| Fig5-fig sup.1B | *DIP-α-T2A-QF2/+;UAS-dpr10-RNAi*/*+; dpr10^1-29^, 10XQUAS-6XGFP* (VK00027)*/ +* |
|  | *DIP-α-T2A-QF2/+;UAS-dpr10-RNAi*/*OK371-Gal4; dpr10^1-29^, 10XQUAS-6XGFP* (VK00027)*/ +* |
| Fig5-fig sup.1C | *DIP-α-T2A-QF2/+; UAS-dpr10-V5* (VK0002)*, 10XQUAS-6XGFP* (VK00018)*/Mef2-Gal4* |
| Fig6A-B,  Fig6-fig sup.1A  Video4-5 | CTRL: *DIP-α-T2A-Gal4/+;; 20XUAS-6XGFP* (attp2), *UAS-DIP-α-V5* (VK00027)/ *20XUAS-6XGFP* (attp2)  Mutant:  *DIP-α-T2A-Gal4/DIP-α^7-1^;; 20XUAS-6XGFP* (attp2)/*20XUAS-6XGFP* (attp2) |
| Fig6-fig sup.1B,  Video3 | *DIP-α-T2A-Gal4/DIP-α^7-1^; VGlut-T2A-QF2, 10XQUAS-6XmCherry (VK00018); 20XUAS-6XGFP* (attp2) |
| Fig7A-B | *DIP-α-T2A-QF2/+;;* *10XQUAS-6XGFP* (attp2) |
| Fig7-fig sup.1A | *DIP-α-T2A-Gal4/DIP-α^7-1^;; 20XUAS-6XGFP* (attp2)/*20XUAS-6XGFP* (attp2) |
| Fig7-fig sup.1B-C | *DIP-α-T2A-Gal4/+;; 20XUAS-6XGFP* (attp2), *UAS-DIP-α-V5* (VK00027)/ *20XUAS-6XGFP* (attp2) |

// - Homozygous
